# Survival in hemodialysis in Brazil according to the source of payment for the treatment: Public Healthcare System (SUS) versus private insurance

**DOI:** 10.1590/2175-8239-JBN-2022-0131en

**Published:** 2023-01-06

**Authors:** Ana Beatriz Lesqueves Barra, Ana Paula Roque da Silva, Maria Eugenia F. Canziani, Jocemir Ronaldo Lugon, Jorge Paulo Strogoff de Matos

**Affiliations:** 1 Universidade Federal Fluminense, Faculdade de Medicina, Programa de Pós-Graduação em Ciências Médicas, Niterói, RJ, Brazil.; 2 Fresenius Medical Care Brasil, Rio de Janeiro, RJ, Brazil.; 3 Universidade Federal de São Paulo, Escola Paulista de Medicina, São Paulo, SP, Brazil.; 4 Universidade Federal Fluminense, Faculdade de Medicina, Divisão de Nefrologia, Niterói, RJ, Brazil.

**Keywords:** Renal Insufficiency, Chronic, Renal Insufficiency; Renal Dialysis, Survival, Brazil, Unified Health System, Insuficiência Renal Crônica, Insuficiência Renal, Diálise Renal, Sobrevida, Brasil, Sistema Único de Saúde

## Abstract

**Introduction:**

Brazil has the largest public and universal healthcare system in the world, but little is known about the outcomes of patients on hemodialysis (HD) in the country according to the source of funding for the treatment.

**Objective:**

To compare the profile and survival of patients under HD treatment funded by the Public Healthcare System (SUS) to those with private insurance.

**Methods:**

Retrospective analysis of adults undergoing HD between 2012 and 2017 in 21 dialysis centers in Brazil that provided both by the SUS and private health insurance. Participants, regardless of the paying source, received similar dialysis treatment. Data were censored after 60 months of follow-up or at the end of 2019.

**Results:**

4,945 patients were included, 59.7% of which were financed by the SUS. Patients financed by SUS, compared to those with private insurance, were younger (58 vs. 60 years; p < 0.0001) and with a lower prevalence of diabetes (35.8% vs. 40.9%; p < 0.0001). The 60-month survival rates in these groups were 51.1% and 52.1%, respectively (p = 0.85). In the analysis of the subdistribution proportional hazard ratio by the Fine-Gray model, including adjustment for concurrent outcomes, a significant increase in the risk ratio for death was found (1.22 [95% confidence interval 1.04 to 1.43]) in patients with treatment funded by the SUS.

**Conclusions:**

Patients on HD with treatment funded by the SUS have a higher adjusted risk of death when compared to those with private insurance, despite similar dialysis treatment. Factors not directly related to dialysis therapy could explain this difference.

## Resumo

**Introdução:**

O Brasil possui o maior sistema público e universal de saúde do mundo, mas pouco se sabe sobre os desfechos dos pacientes em hemodiálise (HD) no país de acordo com a fonte de financiamento do tratamento.

**Objetivo:**

Comparar o perfil e a sobrevida dos pacientes que têm o tratamento de HD custeado pelo Sistema Único de Saúde (SUS) com aqueles com convênio privado. Métodos: Análise retrospectiva dos adultos incidentes em HD entre 2012 e 2017 em 21 centros de diálise no Brasil que atendiam tanto pelo SUS quanto por convênios privados. Os participantes, independentemente da fonte pagadora, receberam tratamento dialítico semelhante. Os dados foram censurados com 60 meses de acompanhamento ou ao final de 2019.

**Resultados:**

Foram incluídos 4945 pacientes, sendo 59,7% financiados pelo SUS. Os pacientes financiados pelo SUS, em comparação aos que tinham convênio privado, eram mais jovens (58 *vs* 60 anos; p < 0,0001) e com menor prevalência de diabetes (35,8% *vs* 40,9%; p < 0,0001). As taxas de sobrevida, em 60 meses nesses grupos foram de 51,1% e 52,1%, respectivamente (p = 0,85). Na análise da razão de risco proporcional de subdistribuição pelo modelo de Fine-Gray, incluindo ajuste para desfechos concorrentes, foi encontrado um aumento significativo na razão de risco para morte (1,22 [intervalo de confiança de 95% 1,04 a 1,43]) nos pacientes com tratamento custeado pelo SUS.

**Conclusões:**

Pacientes em HD com tratamento custeado pelo SUS têm um risco ajustado de morte mais elevado do que aqueles com convênio privado, apesar do tratamento dialítico semelhante. Fatores não relacionados diretamente à terapia dialítica poderiam justificar esta diferença.

## Introduction

The average number of people being treated for functional kidney failure is estimated at 759 per million of the population (pmp) worldwide, with higher rates in high-income countries (969 pmp) compared to upper-middle-income countries (550 pmp), lower-middle (321 pmp), and low-income (4 pmp), despite similar rates of functional renal failure incidence in high-, upper-middle, and lower-middle-income countries (149, 126, and 130 pmp, respectively). This is probably due to the lack of access to treatment in lower-income countries. Public funding for all aspects of renal replacement therapy is available from 75% of high-income countries, 43% of upper-middle-income countries, 19% of lower-middle-income countries, and no low-income countries^1^.

Brazil, an upper-middle-income country, has one of the largest dialysis populations, with an estimated prevalence of 684 pmp, and has the largest universal public healthcare system in the world, the Unified Health System (SUS). SUS was created after the enactment of the 1988 Constitution and has faced the challenge of providing all treatments, including the most complex and with highest financial impact, such as renal replacement therapy^2-4^. It is estimated that Brazil currently has more than 140,000 patients on a dialysis program, around 93% on hemodialysis and 7% on peritoneal dialysis. More than 80% of dialysis patients in the country are financed by the SUS, most of the time in private clinics with contracts. The rest of the patients have their treatments covered by private healthcare insurance, and generally undergo dialysis in the same clinics that treat patients funded by the SUS^4^. The mortality rate among HD patients is extremely high^4-7^. However, little is known about the survival of HD patients in Brazil according to the source paying for the treatment.

Thus, the objective of the present study was to compare the profile and survival of patients who have HD treatment funded by the SUS with those with private insurance.

## Methods

This is a retrospective database analysis of 23 dialysis clinics in Brazil (14 from Rio de Janeiro, 3 from the Federal District, 2 from São Paulo, 2 from Minas Gerais and 2 from Pernambuco). Of these, patients from 2 clinics, one in the Federal District and the other in São Paulo, which exclusively treated patients through private health insurance, were excluded. The remaining 21 clinics treated both SUS and private health insurance patients and were included in the study. All participating clinics used the same electronic medical record, EuCliD^®^ (European Clinical Dialysis Database).

All patients aged 18 years or older undergoing outpatient hemodialysis at participating clinics in the period between July 1, 2012 and June 30, 2017 were included. Patients transferred from other dialysis centers, those who migrated from peritoneal dialysis and those who had previously undergone kidney transplantation were considered prevalent in renal replacement therapy and excluded from the analysis.

The date of the first HD session at the clinic was considered as the start of follow-up. Demographic, clinical and laboratory data on admission were extracted from the EuCliD^®^ in the form of spreadsheets in which the patients were identified only by registration number. Data regarding body composition analyzed by spectroscopic bioimpedance on admission to the clinic were also extracted. An increase in pre-HD extracellular volume above 15% in men or 13% in women was classified as fluid overload^8^.

All the patients received similar dialysis treatment, regardless of the paying source, and in accordance with the country’s legislation^9,10^. The standardized dialyzers were high flux polysulfone or high flux helixone membranes. Automated reuse of dialyzers and blood lines was allowed, except for patients with hepatitis B and C or HIV. All were dialyzed with ultrapure dialysis solution, with glucose 100 mg/dL, potassium 2.0 mEq/L, acetic acid 4 mEq/L and calcium 3.0 mEq/L, but with the option of calcium 2.5 mEq/L at medical discretion. The standard dialysis prescription was sodium 136 mEq/L, bicarbonate 31.5 mEq/L (total buffer 35.5 mEq/L), but with changes in these parameters at the discretion of the attending physician. The standard dialysis solution flow rate was 500 mL/min, temperature 36°C. For patients with arteriovenous fistula, the standard needle was 15G, the blood flow, regardless of the vascular access, was as high as possible, respecting the pressure limits in the arterial and venous lines. The frequency of 3 sessions per week lasting 4 hours was also standardized. Regardless of the paying source, at the discretion of the attending physician, patients with difficulty controlling blood volume could receive a fourth dialysis session in the week for better blood volume control. The only exception was the daily short HD option (5 or 6 times a week, lasting 2 to 3 hours per session), which was basically limited to patients with private insurance, due to lack of coverage by the SUS.

Death from any cause was the main outcome, while kidney transplantation, transfer to peritoneal dialysis and recovery of renal function were considered as concurrent outcomes. The hospitalization rate was also analyzed according to the type of insurance and expressed in number of hospitalizations/patient-year. The data were censored with 5 years of follow-up or on December 31, 2019, to avoid the impact of the covid-19 pandemic on the analysis, and also due to the introduction of online hemodiafiltration from 2020, restricted to patients with private insurance, at one of the participating clinics.

This study was carried out in accordance with the Declaration of Helsinki and was approved by the Research Ethics Committee of the Medical School of the Federal University of Rio de Janeiro, under number CAAE 76623317.1.0000.5243. As it is a retrospective study, using only aggregated data, obtaining the Free and Informed Consent Form was waived by the Ethics Committee.

### Statistical Analysis

The Kolmogorov-Smirnov test was used to test the distribution of variables. Continuous variables with normal distribution were expressed as mean ± standard deviation or as median and interquartile range, otherwise. Categorical variables were presented as frequencies. Comparisons between means in different groups of patients were performed using the unpaired t-test or the Mann-Whitney test. Comparisons between frequencies were performed using the chi-square test. Survival rates were calculated using the Kaplan-Meier method and comparison between the curves was performed using the Log Rank test.

The risk ratios for death were estimated using the subdistribution proportional hazards model described by Fine and Gray^11^, with adjustment for concurrent outcomes (kidney transplantation, migration to peritoneal dialysis and recovery of renal function). Initially, univariate analysis was performed for each variable. Next, only the paying source (SUS) was included in the multivariate analysis as the variable of primary interest and the variables that presented p values < 0.10 in the univariate analysis. Subsequently, the same analysis was performed, but including adjustment for daily HD treatment. Any patient who underwent 20 or more monthly sessions for at least one month during the observation period was considered to have been treated with daily HD. At the end, p values < 0.05 were considered statistically significant. All analyzes were performed using SPSS version 21.0 for Windows (IBM©, Chicago, IL, USA), except for the subdistribution hazard ratio analysis using the Fine-Gray method, which was performed using the freely available R software version 4.0.2.

## Results

Initially there were 5,129 patients undergoing HD in the period, but after excluding 136 patients from the two clinics not affiliated with the SUS and 48 aged under 18 years, a total of 4,945 patients were included in the analysis (59.3% were men, 37.5% had diabetes as the cause of renal failure, 29.8% had arteriovenous fistula as initial vascular access, and 60.2% started HD in hospital). The characteristics of the patients upon admission, as well as the comparisons between those with treatment funded by the SUS or by private health plans, are shown in Table 1.

**Table 1.** Data of all the patients on HD upon admission and according to the treatment’s paying source

Characteristics	All (n = 4,945)	SUS (n = 2,951)	Private (n = 1,994)	p-value*
Males, n (%)	2,932 (59.3)	1,713 (58.0)	1,219 (61.1)	0.033
Age, years	59 (47 – 69)	58 (46 – 67)	60 (48 – 71)	<0.0001
Age ≥ 65 years, n (%)	1,727 (34.9)	923 (31.3)	804 (40.3)	<0.0001
Skin color not white, n (%)	2,823 (57.1)	1,877 (63.6)	946 (47.4)	<0.0001
Renal failure cause, n (%)				
Diabetes	1,856 (37.5)	1,040 (35.8)	816 (40.9)	<0.0001
Hypertension	1,288 (26.0)	837 (28.4)	451 (22.6)	<0.0001
Glomerulonephritis	532 (10.8)	337 (11.4)	195 (9.8)	0.075
Kidney disease polycystic	186 (3.8)	97 (3.3)	89 (4.5)	0.040
Others	340 (6.9)	182 (6.2)	158 (7.9)	0.019
Undetermined	743 (15.0)	459 (15.6)	284 (14.2)	0.22
Prior follow-up, n (%)	1,912 (38.7)	1,057 (35.8)	855 (42.9)	<0.0001
Place of first dialysis				
Hospital	2,978 (60.2)	1,707 (57.9)	1,271 (63.7)	<0.0001
Dialysis clinic	1,283 (25.9)	817 (27.7)	466 (23.4)	0.0008
No information	684 (13.8)	427 (14.5)	257 (12.9)	0.12
Initial vascular access n (%)				
Active AVF	1,474 (29.8)	933 (31.6)	541 (27.1)	0.0008
Graft	33 (0.7)	21 (0.7)	12 (0.6)	0.77
Temporary Catheter	2,801 (56.6)	1,815 (61.5)	986 (49.4)	<0.0001
Tunnel Catheter	637 (12.9)	182 (6.2)	455 (22.8)	<0.0001
B Hepatitis, n (%)	33 (0.7)	24 (0.8)	9 (0.5)	0.18
C Hepatitis, n (%)	131 (2.6)	87 (2.9)	44 (2.2)	0.13
HIV Infection, n (%)	43 (0.9)	24 (0.8)	19 (1.0)	0.72
Erythropoietin use, n (%)	2,140 (43.3)	1,278 (43.3)	862 (43.2)	0.98
Hemoglobin, g/dL	9.8 (8.3 – 11.3)	9.8 (8.2 – 11.4)	9.8 (8.5 – 11.1)	0.96
Transferrin saturation, %	24 (17 – 35)	25 (17 – 36)	24 (16 – 32)	<0.0001
Ferritin, ng/mL	364 (155 – 724)	390 (170 – 773)	318 (133 – 649)	<0.0001
Pre-HD urea, mg/dL	116 (91 – 147)	122 (96 – 154)	115 (90 – 144)	0.018
Albumin, g/L	36 (33 – 40)	36 (32 – 39)	37 (33 – 40)	<0.0001
Potassium, mEq/L	5.1 (4.5 – 5.8)	5.1 (4.5 – 5.8)	5.0 (4.5 – 5.7)	0.17
Phosphorus, mg/dL	4.6 (3.7 – 5.7)	4.6 (3.7 – 5.7)	4.5 (3.6 – 5.6)	0.21
Calcium corrected, mg/L	8.9 (8.3 – 9.4)	9.0 (8.5 – 9.5)	8.9 (8.3 – 9.4)	0.03
PTHi, pg/mL	265 (126 – 515)	305 (150 – 575)	214 (100 – 417)	<0.0001
Alkaline phosphatase, UI/L	96 (73 – 137)	99 (75 – 143)	92 (70 – 127)	0.0003
SBP pre-HD, mmHg	142 (129 – 156)	143 (130 – 157)	140 (128 – 155)	<0.0001
DBP pre-HD, mmHg	79 (71 – 85)	80 (73 – 87)	77 (68 – 83)	<0.0001
BMI, Kg/m^2^	23.7 (21.0 – 27.0)	23.4 (20.8 – 26.4)	24.2 (21.4 – 27.6)	<0.0001
Spectroscopic bioimpedance				
Lean mass, %	49.4 (39.9 – 60.4)	51.0 (41.1 – 62.3)	47.6 (38.2 – 57.7)	<0.0001
Fat tissue, %	34.0 (25.5 – 41.3)	32.9 (24.3 – 40.2)	35.5 (27.6 – 42.8)	<0.0001
Excessive ECV, %	12.3 (4.5 – 20.7)	12.9 (4.7 – 21.1)	11.8 (4.5 – 20.5)	0.44
Fluid overload n (%)	1,664 (45.2)	997 (44.9)	667 (45.7)	0.62

^*^Private *vs.* SUS; SUS: Public Healthcare System; AVF: arteriovenous fistula; HIV: Human immunodeficiency virus; PTHi: intact parathyroid hormone; SBP: systolic blood pressure; DBP: diastolic blood pressure; BMI: body mass index; ECV: extracellular volume. Values expressed by frequency or median (interquartile interval).

Almost 60% of the patients were financed by the SUS and the rest by private insurance. Patients with private insurance, compared to those financed by the SUS, were older (60 vs. 58 years; p < 0.0001), with a higher prevalence of diabetes as a cause of renal failure (40.9% vs. 35 .8%; p < 0.0001) and, more frequently, had been followed up by a nephrologist before starting dialysis (42.9% vs. 35.8%; p < 0.0001), although a higher percentage of them had unplanned HD started in hospital (63.7% vs. 57.9%; p < 0.0001). Patients whose treatment was funded by the SUS more frequently started HD through AVF (31.6% vs. 27.1%; p = 0.0008), had more temporary catheters and less tunneled catheters (6.2% vs. 22, 8%; p < 0.0001) as initial vascular access. Of the 3,682 patients assessed by bioimpedance on admission, 45.2% had fluid overload, but with no difference between groups. These and other comparisons between the two groups are shown in Table 1.

During the study period, 1,605 patients died, 1,037 were transferred to other centers, 511 underwent kidney transplantation, 243 migrated to peritoneal dialysis, 238 recovered kidney function and 49 lost follow-up. The mean follow-up time was 26.7 months. HD exits due to kidney transplantation (12.9% vs. 9.9%; p = 0.001) and recovery of renal function (6.1% vs. 3.9%; p = 0.0005) were more frequent among patients with private insurance, but not switching to peritoneal dialysis (4.7% vs. 5.0%; p = 0.64).

Over the follow-up period, the hospitalization rate was higher among patients with private insurance than among those with SUS-funded treatment (1.02 vs. 0.43 hospitalization/patient-year, p < 0.0001) .

During the follow-up period, 355 patients with private insurance (17.8%) and 8 with treatment funded by the SUS (0.3%) were treated with daily short HD for at least one month. The median and interquartile range of time that patients were on daily HD was 9 (5 to 19) months.

**Figure 1.**
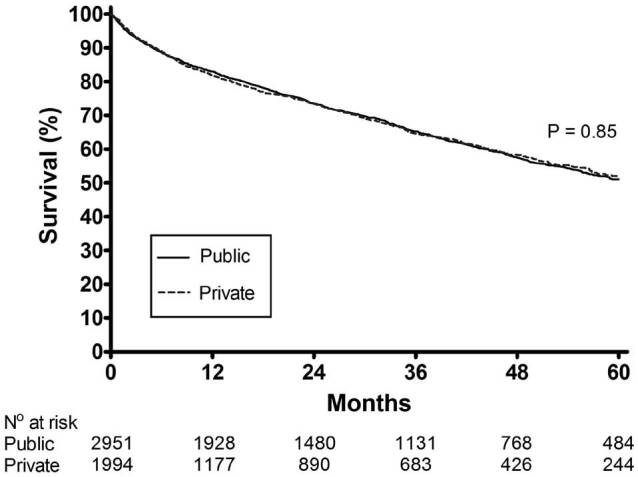
Survival curves, according to the paying source for the treatment. SUS, Brazilian Public Healthcare system.

Survival rates, by Kaplan-Meier curves, at 60 months among patients with private insurance or with treatment funded by SUS were 52.1% and 51.1%, respectively (p = 0.85), Figure 1 In the univariate analysis of the subdistribution using the Fine-Gray model, with kidney transplantation, transfer to peritoneal dialysis and recovery of renal function as concurrent outcomes for death, the proportional hazard ratio of the death of patients who had treatment funded by the SUS compared with patients with private insurance was 1.08 (95%CI 0.93 to 1.19). Next, the same subdistribution proportional hazard ratio analysis was performed, but with adjustment for all independent variables that had a p-value < 0.10 in the univariate analysis. In this adjusted model, a significant increase in the risk ratio for death in patients treated by the SUS of 1.22 (95% confidence interval 1.04 to 1.43) was found. After adjusting for daily short HD treatment, the risk ratio for death in patients with treatment funded by the SUS remained high (1.24, with a 95% confidence interval 1.05 to 1.47).

Other variables associated with increased risk of death in the adjusted model were age (p < 0.0001), diabetes (p < 0.0001), initiation of HD in hospital (p = 0.0057), preoperative fluid overload -dialysis by bioimpedance (p < 0.0001) and higher levels of alkaline phosphatase (p = 0.0083), while the body mass index (p = 0.014), hemoglobin levels (p = 0.0074), serum albumin (p = 0.0001), and higher transferrin saturation index (p = 0.0063) were associated with a lower risk of death. The associations between gender, previous follow-up with a nephrologist, the beginning of HD via catheter, serum levels of phosphorus, calcium, parathyroid hormone, lean mass index and pre-HD diastolic blood pressure with the risk of death, found in the analyzes univariate, disappeared in the adjusted model (Table 2).

**Table 2.** Underdistribution risk ratio by the Fine-Gray method to analyze the risk of death during the 60 months of follow-up, according to patients’ characteristics upon admission

	Non-adjusted hazard ratio (CI 95%)	p-value	Adjusted hazard ratio (CI 95%)	P-value
Dialysis by the SUS	1.08 (0.93 – 1.19)	0.13	1.22 (1.04 – 1.43)	0.013
Males	0.85 (0.77 – 0.93)	0.0008	0.97 (0.84 – 1.14)	0.74
Age (years)	1.04 (1.03 – 1.04)	<0.0001	1.03 (1.03 – 1.04)	<0.0001
Non-white skin color	0.96 (0.87 – 1.06)	0.41	–	–
Diabetes	1.31 (1.19 – 1.45)	<0.0001	1.29 (1.10 – 1.51)	0.0018
Followed up by nephrologist	0.72 (0.65 – 0.81)	<0.0001	0.90 (0.74 – 1.09)	0.28
First hospital HD	1.56 (1.38 – 1.77)	<0.0001	1.35 (1.09 – 1.68)	0.0057
Catheter	1.76 (1.57 – 1.97)	<0.0001	1.12 (0.95 – 1.32)	0.19
BMI (Kg/m^2^)	0.97 (0.96 – 0.98)	<0.0001	0.98 (0.96 – 0.99)	0.014
Hemoglobin (g/dL)	0.88 (0.86 – 0.91)	<0.0001	0.95 (0.92 – 0.99)	0.0074
TSI ≥ 20%	0.70 (0.63 – 0.78)	<0.0001	0.81 (0.70 – 0.94)	0.0063
Ferritin ≥ 200 ng/mL	1.07 (0.96 – 1.20)	0.21	–	–
Erythropoietin use	0.97 (0.88 – 1.07)	0.56	–	–
Urea (mg/dL)	0.99 (0.99 – 0.99)	<0.0001	1.00 (0.99 – 1.00)	0.30
Serum albumin (g/L)	0.93 (0.92 – 0.94)	<0.0001	0.97 (0.95 – 0.98)	0.0001
Potassium (mEq/L)	1.01 (0.96 – 1.07)	0.59	–	–
Phosphorus (mg/dL)	0.90 (0.87 – 0.93)	<0.0001	1.00 (0.95 – 1.06)	0.99
Corrected calcium (mg/L)	1.03 (1.00 – 1.07)	0.042	1.01 (0.96 – 1.06)	0.72
PTHi (per 100 pg/mL)	0.96 (0.95 – 0.98)	<0.0001	1.00 (0.98 – 1.02)	0.72
Alkaline phosphatase (per 100 UI/L)	1.09 (1.06 – 1.12)	<0.0001	1.07 (1.02 – 1.13)	0.0083
SBP pre-HD (mmHg)	1.00 (1.00 – 1.00)	0.62	–	–
DBP pre-HD (mmHg)	0.99 (0.98 – 0.99)	<0.0001	1.00 (0.99 – 1.01)	0.80
Lean mass (%)	0.99 (0.98 – 0.99)	<0.0001	0.99 (0.99 – 1.00)	0.083
Fat tissue (%)	1.00 (1.00 – 1.01)	0.28	–	–
Fluid overload	1.95 (1.73 – 2.20)	<0.0001	1.53 (1.31 – 1.78)	<0.0001

CI: confidence interval; BMI: body mass index; TSI: transferrin saturation index; PTHi: intact parathyroid hormone; SBP: systolic blood pressure; DBP: diastolic blood pressure.

## Discussion

The present study made it possible to compare the profile of patients who entered an HD program with treatment funded by the SUS, with that of patients using private healthcare insurance treated at the same clinics. It was possible to study the survival of these patients over a period of up to 5 years and define the association between the type of coverage of dialysis treatment costs and the risk of death. As far as we know, this is the first study of this nature in the country and its findings could contribute to the development of healthcare policies aimed at improving medical care and survival of patients on HD with treatment funded by the SUS.

The 5-year survival rates were similar. However, as patients with treatment funded by the SUS, among other differences, were younger and had a lower prevalence of diabetes than patients with private insurance, a 22% increase in the adjusted risk of death.

The reasons for this increase in the risk of death among patients using the SUS are open to debate. All adjustments in the risk of death analysis were initially made for the characteristics of the patients upon admission, but not for how they were treated over the follow-up period. However, it is unlikely that differences in the dialysis treatment provided to patients, according to the paying source, can explain the difference in outcomes, since, regardless of the paying source, all received very similar treatments, including dialysis machines, dialyzers, dialysis solution, prescription dialysis, and were assisted by the same nephrologists. The only difference in treatment may have been the availability of the daily short HD option for privately insured patients. However, the vast majority of patients, even with private insurance, spent the entire follow-up period on the traditional HD scheme, with 3 weekly sessions. In any case, seeking to adjust for an effect of this concerning dialysis on survival, an additional analysis was performed, including adjustment for daily short HD exposure. Even after this adjustment, the risk of death among patients whose treatment was funded by the SUS changed little, remaining significantly higher.

The most plausible justification for the difference in the risk of death according to the source of payment for the treatment would be that patients with private insurance had easier access to diagnostic tests, follow-up and treatment by other specialties, as well as extra dialytic medications, although these variables were not evaluated in the present study. The hospitalization rate among patients with private insurance, which was more than twice that observed among patients assisted by the SUS, may reflect the difference in access to medical care in general. Despite universal access to the public healthcare system in Brazil, there is still inefficiency in hospital care, with inequalities in access and in the adequacy between demand and supply of vacancies, heterogeneity in the quality of services and deficiency in the integration of hospitals in the care network^2,12,13^.

As demonstrated in the present study, patients dependent on the SUS have a disadvantage in the risk of death even when receiving similar dialysis treatment. It is possible that this situation will get worse as a result of insufficient funding of renal replacement therapy by the SUS over the years, which may provide conditions for widening the gap in the quality of dialysis and, even, in access to dialysis treatment in the country. The lag in reimbursement values has inhibited the expansion of SUS-accredited places in private dialysis clinics in the country and led to the opening of new dialysis centers aimed exclusively at patients with private insurance, mainly in the country’s large cities. In 2018, the average estimated reimbursement value per HD session by private healthcare insurance was approximately double than that reimbursed by the SUS (105.00 and 53.00 US dollars, respectively)^14^. More recently, after the inclusion of online hemodiafiltration in the list of therapies authorized by the National Agency for Supplementary Health^15^, an increasing number of patients with private healthcare insurance have been treated by this modality of dialysis and with a single use of dialyzers, widening the difference in the way they are cared for depending on the funding source of the treatment.

The other admission variables associated with the risk of death found in the present study were age, diabetes, initiation of HD in a hospital, body mass index, transferrin saturation index, hemoglobin, albumin and alkaline phosphatase levels and fluid overload (Table 2). The association between these variables and the risk of death are well known^8,16-21^ and are not the focus of the present study, as we have already analyzed and discussed such associations in more detail in a previous study with the same cohort^7^.

The present study has several limitations, including the retrospective nature of the analysis, the very high number of clinics in the state of Rio de Janeiro, the significant number of patients without bioimpedance evaluation, and some differences in the list of routine laboratory tests performed. While all patients had a list of laboratory tests and the periodicity of their performance in accordance with legal requirements^9,10^, patients with private insurance also had some tests not covered by the SUS, such as bicarbonate dosage, hemoglobin A1c, β2- microglobulin and C-reactive protein. On the other hand, the study also has its strengths, such as the large sample size and the long follow-up period.

In conclusion, patients on HD with treatment funded by the SUS have a higher adjusted risk of death than those with private insurance, despite similar dialysis treatment. Factors not directly related to dialysis, such as greater access to diagnostic tests, procedures and hospitalization among patients with private insurance could explain this difference. The identification of these factors, as well as the planning and implementation of public policies aimed at improving and enhancing this population’s access to medical care more broadly in the SUS network, going well beyond the right to dialysis treatment, could have an effect favorable in reducing mortality.

## References

[1] Yeung E, Bello AK, Levin A, Lunney M, Osman MA, Ye F (2021). Current status of health systems financing and oversight for
end-stage kidney disease care: a cross-sectional global
survey. BMJ Open.

[2] Castro MC, Massuda A, Almeida G, Menezes-Filho NA, Andrade MV, de Souza Noronha KVM (2019). Brazil’s unified health system: the first 30 years and prospects
for the future. Lancet.

[3] Alcalde PR, Kirsztajn GM (2018). Expenses of the Brazilian Public Healthcare System with chronic
kidney disease. J Bras Nefrol.

[4] Nerbass FB, Lima HDN, Thomé FS, Vieira OM, Lugon JR, Sesso R (2022). Brazilian Dialysis Survey 2020. J Bras Nefrol.

[5] United States Renal Data System (2020). 2020 USRDS annual data report: Epidemiology of kidney disease in the
United States.

[6] ERA-EDTA (2021). ERA-EDTA Registry: Annual Report 2019.

[7] Barra ABL, Roque-da-Silva AP, Canziani MEF, Lugon JR, Strogoff-de-Matos JP (2022). Characteristics and predictors of mortality on haemodialysis in
Brazil: a cohort of 5,081 incident patients.. BMC Nephrol.

[8] Zoccali C, Moissl U, Chazot C, Mallamaci F, Tripepi G, Arkossy O (2017). Chonic Fluid overload and mortality in ESRD. J Am Soc Nephrol.

[9] Brasil (2011). Ministério da Saúde. Agência Nacional de Vigilância Sanitária. Resolução
de Diretoria Colegiada - RDC nº 6, de 14 de fevereiro de 2011. Altera a
Resolução RDC n. 154, de 15 de junho de 2004, que estabelece o Regulamento
Técnico para o funcionamento dos Serviços de Diálise, republicada em
31/05/2006.

[10] Brasil (2014). Ministério da Saúde. Agência Nacional de Vigilância Sanitária. Resolução
da Diretoria Colegiada - RDC nº 11, de 13 de março de 2014. Dispõe sobre os
Requisitos de Boas Práticas de Funcionamento para os Serviços de Diálise e
dá outras providências.

[11] Fine JP, Gray RJ (1999). A proportional hazards model for the subdistribution of a
competing risk. J Am Stat Assoc.

[12] de Almeida Botega L, Andrade MV, Guedes GR (2020). Brazilian hospitals’ performance: an assessment of the unified
health system (SUS). Health Care Manag Sci.

[13] Botega LA, Andrade MV, Guedes GR (2020). Profile of general hospitals in the Unified Health
System. Rev Saude Publica.

[14] Sesso R, Lugon JR (2020). Global Dialysis Perspective: Brazil. Kidney360.

[15] Agência Nacional de Saúde Suplementar (2021). Rol de Procedimentos e Eventos em Saúde, Resolução Normativa nº 465 de
24 de fevereiro de 2021, publicada em 02/03/2021.

[16] Bradbury BD, Fissell RB, Albert JM, Anthony MS, Critchlow CW, Pisoni RL (2007). Predictors of early mortality among incident US hemodialysis
patients in the Dialysis Outcomes and Practice Patterns Study
(DOPPS). Clin J Am Soc Nephrol.

[17] Owen Jr WF, Lew NL, Liu Y, Lowrie EG, Lazarus JM (1993). The urea reduction ratio and serum albumin concentration as
predictors of mortality in patients undergoing hemodialysis. N Engl J Med.

[18] Sumida K, Molnar MZ, Potukuchi PK, Thomas F, Lu JL, Obi Y (2018). Prognostic significance of pre-end-stage renal disease serum
alkaline phosphatase for post-end-stage renal disease mortality in
late-stage chronic kidney disease patients transitioning to
dialysis. Nephrol Dial Transplant.

[19] Guedes M, Muenz DG, Zee J, Bieber B, Stengel B, Massy ZA (2021). Serum biomarkers of iron stores are associated with increased
risk of all-cause mortality and cardiovascular events in nondialysis CKD
patients, with or without anemia. J Am Soc Nephrol.

[20] Kuragano T, Joki N, Hase H, Kitamura K, Murata T, Fujimoto S (2020). Low transferrin saturation (TSAT) and high ferritin levels are
significant predictors for cerebrovascular and cardiovascular disease and
death in maintenance hemodialysis patients. PLoS One.

[21] Sato M, Hanafusa N, Tsuchiya K, Kawaguchi H, Nitta K (2019). Impact of transferrin saturation on all-cause mortality in
patients on maintenance hemodialysis. Blood Purif.

